# Effect of Alkaline Earth Metal on AZrO_x_ (A = Mg, Sr, Ba) Memory Application

**DOI:** 10.3390/gels8010020

**Published:** 2021-12-27

**Authors:** Ke-Jing Lee, Yeong-Her Wang

**Affiliations:** Department of Electrical Engineering, Institute of Microelectronics, National Cheng-Kung University, Tainan 701, Taiwan; Luke.k.j.lee@gmail.com

**Keywords:** alkaline earth metal, resistive switching, memory, oxygen vacancy, sol–gel

## Abstract

Zr can be stabilized by the element selected, such as Mg-stabilized Zr (MSZ), thus providing MSZ thin films with potentially wide applications and outstanding properties. This work employed the element from alkaline earth metal stabilized Zr to investigate the electrical properties of sol–gel AZrO_x_ (A = alkaline earth metal; Mg, Sr, Ba) as dielectric layer in metal-insulator–metal resistive random-access memory devices. In addition, the Hume–Rothery rule was used to calculate the different atomic radii of elements. The results show that the hydrolyzed particles, surface roughness, and density of oxygen vacancy decreased with decreased difference in atomic radius between Zr and alkaline earth metal. The MgZrOx (MZO) thin film has fewer particles, smoother surface, and less density of oxygen vacancy than the SrZrO_x_ (SZO) and BaZrO_x_ (BZO) thin films, leading to the lower high resistance state (HRS) current and higher ON/OFF ratio. Thus, a suitable element selection for the sol–gel AZrO_x_ memory devices is helpful for reducing the HRS current and improving the ON/OFF ratio. These results were obtained possibly because Mg has a similar atomic radius as Zr and the MgO_x_-stabilized ZrO_x_.

## 1. Introduction

Metal oxide materials for resistive random-access memory (RRAM) have gained considerable attention in recent years. Zirconium dioxide (ZrO_2_) is one of the most studied metal oxide materials [1,2,3,4]. It was reported that the resistive switching characteristics of ZrO_2_-based RRAM devices can be further improved by doping of different impurities [5]. Addition of promoters, such as magnesium oxide (MgO), yttrium oxide (Y_2_O_3_), and calcium oxide (CaO) is also reported to benefit the catalyst stability [6]. The incorporation of other elements, such as Mg, in ZrO_2_ thin films may improve the RRAM. BaZrO_3_ and SrZrO_3_ have cubic and slightly distorted perovskite structures with lattice constants of 0.419 and 0.409 nm, respectively. Because of reported dielectric constants of 15 and chemical stability [7,8]. The BaZrO_3_ and SrZrO_3_ films exhibited clear interfaces and smooth surfaces. When BaZrO_3_ films contained excess Ba, the film surface is rough and poor adhesion [9]. Porter et al. reported the carrier consisting of inorganic oxide preferably contains zirconia stabilized by the element selected from Y, Ce, Mg, Sc, and Sm, such as yttria-stabilized zirconia [10]. The compositions of ZrO_2_, MgO, or CaO-stabilized zirconia (MSZ or CSZ) play an important role owing to their high mechanical strength, good chemical stability, high level of oxygen-ion conductivity, corrosion resistance, and low thermal conductivity, thus allowing its wide applications. According to our previous work, the density of hydrolytic particles, surface roughness, and oxygen vacancies all decrease when the difference in atomic radii between Ti and alkaline earth oxide based memory devices favors a reduction in the current in the high reluctance state (HRS) [11].

Thus, if the RRAM consisting of various metal oxide-stabilized Zr can be easily pre-pared through solution process or sol–gel method has the advantages of low cost, easy stoichiometric control, high uniformity, and reversible resistance switching properties, such an approach can provide an essential basis for the fabrication of RRAM devices with simplified manufacturing process [5,6,7,8,9,10,11,12].

The sol–gel metal oxide-stabilized Zr (or AZrO_x_, A = alkaline earth metal) with multi-cations contain complex chemical compositions compared with the binary transition metal oxide, and the different alkaline earth metal stabilized-Zr can effectively different properties of thin film, such as the density of particles, pits, oxygen vacancies, and surface roughness, thus affecting the electrical properties [13,14]. Furthermore, based on the Hume–Rothery rules, when the atomic radius of the solute and solvent atoms differs by more than 15%, phase separation may occur. This phenomenon indicates that the different atomic radii of elements cause the different surface morphology of the AZrO_x_ thin film.

Therefore, solution-processed materials that allow easy control of the chemical composition and selection of suitable alkaline earth metals are needed to synthesize the resistive layer of RRAM devices. In this work, the relationship between element (alkaline earth metal) selection and memory properties in AZrO_x_-based RRAM applications is investigated.

## 2. Results and Discussion

**Figure 1:**
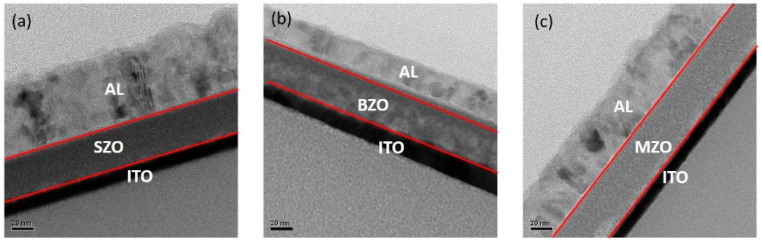
Cross-sectional TEM images of Al/(**a**) strontium zirconate (SZO), (**b**) barium zirconate (BZO), and (**c**) magnesium zirconate (MZO) /ITO/glass devices.

**Figure 2:**
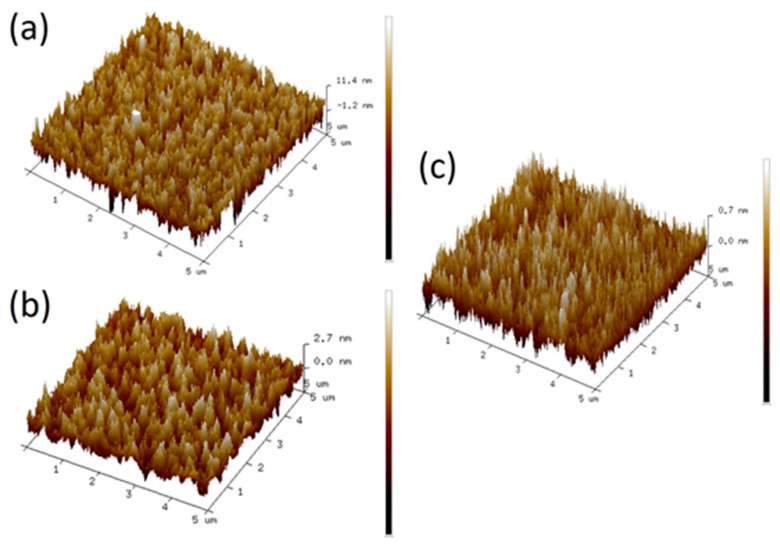
The surface morphologies at (**a**) SZO, (**b**) BZO, and (**c**) MZO thin films for AFM images.

Figure 1 shows the cross-sectional high-resolution transmission electron microscope (TEM) images of the Al/(a)strontium zirconate (SZO), (b)barium zirconate (BZO), and (c)magnesium zirconate (MZO) /ITO/glass structure. The bottom electrodes consist of 10 nm-thick ITO, and the top electrodes consist of 55 nm-thick Al. The cross-sectional TEM image indicates that the thickness of the AZO_x_ film is approximately 35 nm. Figure 2 displays the Atomic Force Microscope (AFM) images of the surface morphologies at SZO, BZO, and MZO thin film. The root mean squared surface roughness (R*_rms_*) values of the SZO, BZO, and MZO thin films on the ITO/Glass substrate were approximately 2.96, 1.91, and 0.14 nm, respectively. The surface of the SZO thin film was rough, whereas the MZO thin film has nearly neither particles nor pits in the surface. Thus, selecting alkaline earth metal materials will help achieve better surface appearance and smooth surfaces.

**Figure 3:**
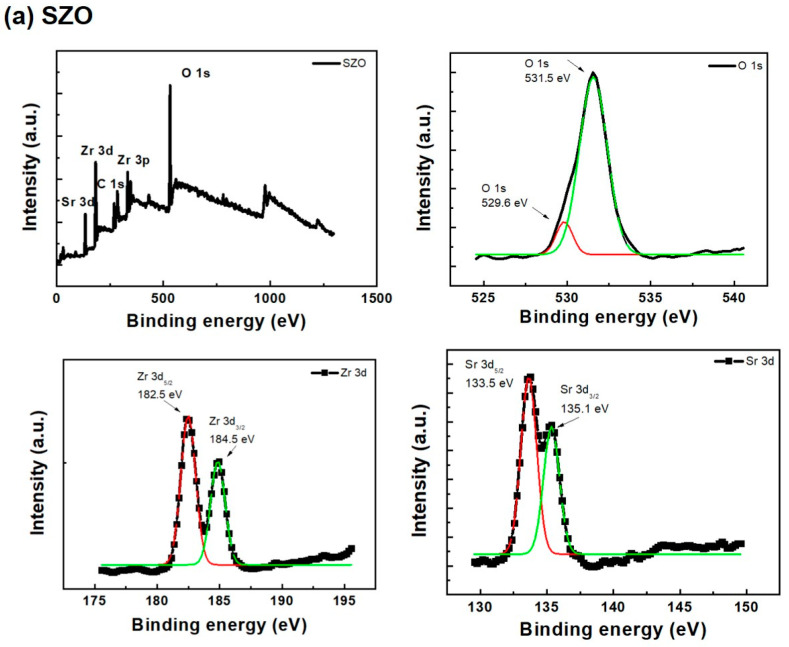

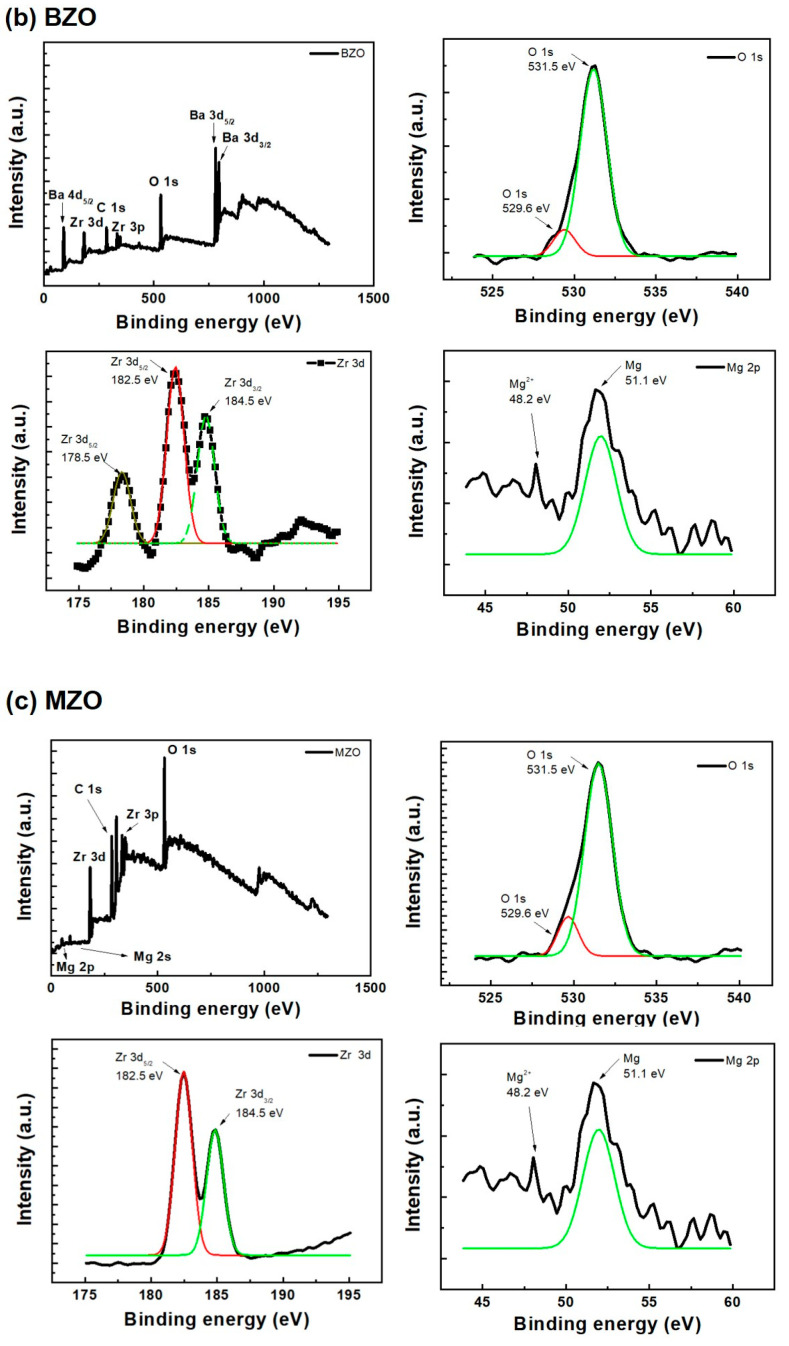
XPS full spectra of (**a**) SZO, (**b**) BZO, and (**c**) MZO thin films.

The experiment step was similar with our previous work [15,16,17,18], in which the SZO, BZO, and MZO thin films are amorphous. Figure 3 shows the elements present in each sample are clearly identified. The samples contain the elements zirconium, oxygen, magnesium, strontium, barium and carbon. The carbon present in the sample prepared in water is from solution the containing hydrocarbon and was removed by Ar^+^ bombardment. Its binding energy is at 284.6 eV. The highly contained carbon in the thin films may have impact in the devise performance, especially in the reliability [19,20,21,22]. Carbon is not discussed here. Two main peaks’ position Zr 3d_5/2_ and Zr 3d_3/2_ are to be found at 182.5 eV and 184.5 eV, respectively: hence, the form of zirconium was Zr^4+^ in zirconium dioxide. The Mg 2P shows 48.2 eV associated with Mg (0) and 51.1 eV associated with Mg^2+^ corresponding to magnesium oxide (MgO), respectively. This result indicates the presence of Mg^2+^ valence states, typically can be attributed to the presence of Mg^2+^ replacing Zr^4+^ lattice.

The peaks for the O1s core level may be consistently fitted by two different near–Gaussian subpeaks centered at and 529.6 and 531.5 eV. The binding energy of lattice oxygen is 529.6 eV, which is attributed to the binding of O_2_ ions to metal ions. The peak at 531.5 eV is associated with non-lattice oxygen source ions, such as oxygen vacancies. The devices were operated without a forming process because of the sufficient non–lattice oxygen ions in the SZO, BZO and the MZO thin films. According to the XPS results, the O_vacancy_/O_lattice_ ratio, where O_lattice_ refers to the lattice oxygen, can be considered as a criterion for comparison. The ratios of O_vacancy_/O_lattice_ in the XPS spectra are approximately 2.38, 2.1, and 1.75 for SZO, BZO, and MZO, respectively. The HRS current value of memory device increased with increasing density of oxygen vacancy [23]. Huang et al. reported that the amount of filament paths is proportional to the density of oxygen vacancies [24]. The large oxygen vacancies can form the filament paths easier. However, excess oxygen vacancies will lead to the sudden formation of numerous filament paths and further affect the performance and stability of the memory devices in amorphous thin film. Thus, the HRS currents and reset voltages of BZO/SZO memory devices were relatively higher than those of MZO memory device. The XPS results show the trend of oxygen vacancy and further explain the lower HRS current or higher ON/OFF ratio of MZO-based RRAM.

**Figure 4:**
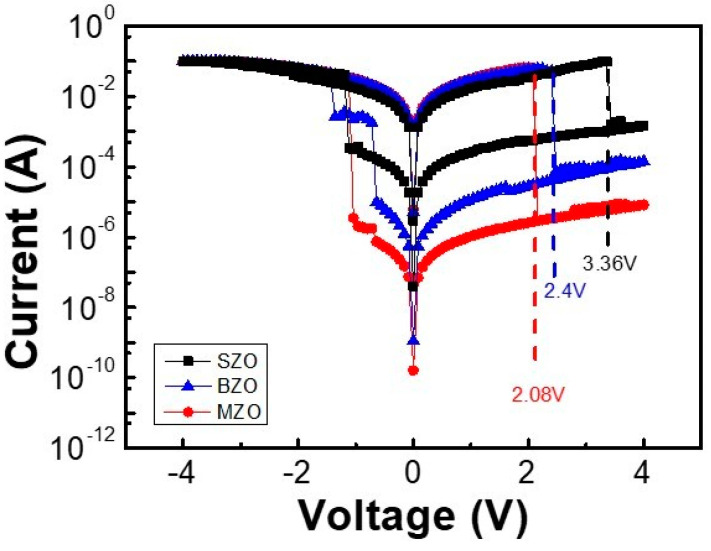
Typical I–V characteristic of AZO_x_-based RRAM plotted on a semi-logarithmic scale. (A = Sr, Ba, Mg).

The typical I–V curve of the SZO, BZO, and MZO-based RRAMs are presented in Figure 4. These devices operate without a forming process due to the presence of sufficient amorphous oxygen ions in the film [11]. Depending on the element selection the HRS values are different [25,26,27]. Both the reset voltage and HRS current values gradually increased with the atomic number of IIa group. The HRS current values of the SZO, BZO, and MZO memory device were 1.8 × 10^−5^, 5.4 × 10^−7^, and 7.1 × 10^−8^ A, respectively. The reset voltages values of the SZO, and BZO memory device were 3.36 V, and 2.4 V, respectively. The set voltages values of the SZO, and BZO memory device were −1.12 V, and −1.36 V, respectively. The Al/MZO/ITO device set and reset voltages of −1.04 and 2.08 V, respectively.

The details of Hume–Rothery rules were introduced in previously work [11]. The radii of Mg^2+^ and Zr^4+^ ion (or Mg and Zr atom) are almost the same (160, 86, 160, and 86 pm for Mg, Mg^2+^, Zr, and Zr^2+^, respectively). The electronegativities of Mg and Zr atoms on the Pauling scale are close (Mg: 1.31, Zr: 1.33). Based on the Hume–Rothery rules, the difference in the atomic radius of Sr, Ba, and Mg between Zr atoms were 34, 35, and 0%, respectively. Considering that the atomic radius of Sr/Ba and Zr atoms differ by more than 30%, phase separation may occur in the SZO and BZO thin film. In addition, considering that Sr (Sr: 0.95) and Ba (Ba: 0.89) has electronegativity, Sr/Ba prevented Zr from combining with oxygen during deposition. This phenomenon leads to the generation of higher density of oxygen vacancy observed for the SZO and BZO thin films.

**Figure 5:**
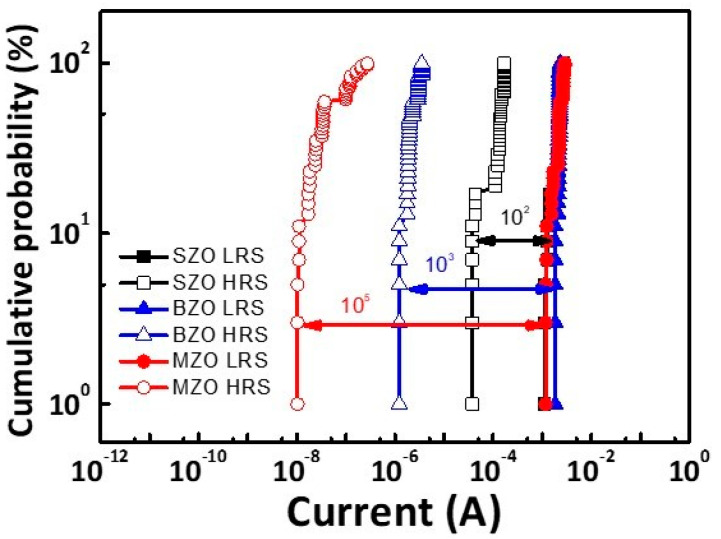
Cumulative distributions of current values for Al/SZO/ITO, Al/BZO/ITO, and Al/MZO/ITO devices measured at 0.5 V.

Figure 5 shows the Cumulative distributions of low resistance state (LRS) and HRS resistances for over 100 devices. Considering different means, the coefficient of variation (CV) is used to evaluate the probability distribution, which is defined as the ratio of the standard deviation (σ) to the mean (μ). The current SZO CV values for LRS and HRS were 27% and 36%, respectively. The current BZO CV values for LRS and HRS were 8% and 48%, respectively. The current MZO CV values for LRS and HRS were 23% and 96%, respectively. The ON/OFF ratio of the SZO, BZO, and MZO memory device were 10^2^, 10^3^, and 10^5^, respectively. The larger CV distribution for LRS than for HRS was associated with the higher current in the LRS that formed the current conduction path.

**Figure 6:**
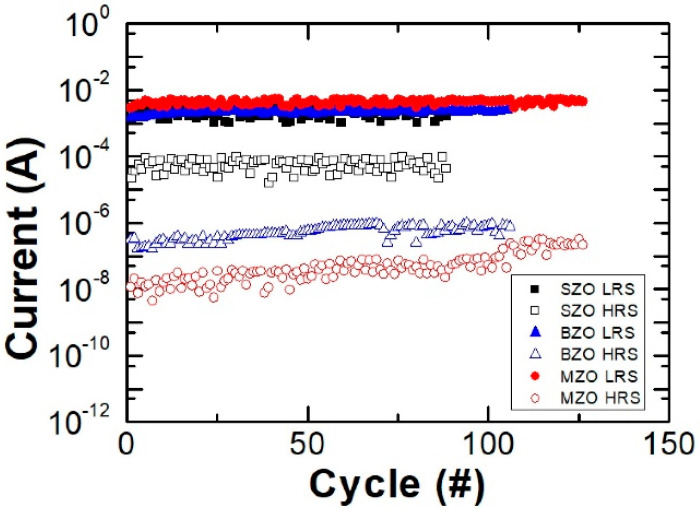
Endurance performance of Al/SZO/ITO, Al/BZO/ITO, and Al/MZO/ITO RRAM operated under DC bias mode at RT.

The endurance behaveiors of SZO, BZO, and MZO thin film memories under DC sweep mode at room temperature are shown in Figure 6. The resistive switching behavior patterns are all stably repeated for over 100 cycles at a reading voltage of 0.5 V.

**Figure 7:**
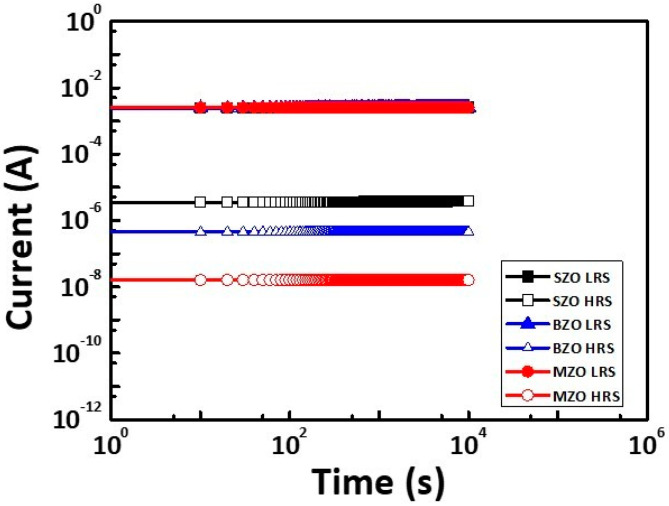
Retention test for the ON and OFF state for Al/SZO/ITO, Al/BZO/ITO, and Al/MZO/ITO RRAM at RT.

Figure 7 shows the retention characteristics of SZO, BZO, and MZO thin film at room temperature (RT). Retention time in RT of longer than 10^5^ s was observed. The ON/OFF ratio of 10^5^ for MZO devise can be deduced to be maintained for the next 10 years. Accordingly, the MZO thin film has smooth thin film, less particle, and less oxygen vacancy. Thus, the MZO memory device has the lowest HRS current and highest ON/OFF ratio. These results were obtained possibly because Mg has a similar atomic radius as Zr and the MgO_x_-stablized ZrO_x_.

**Figure 8:**
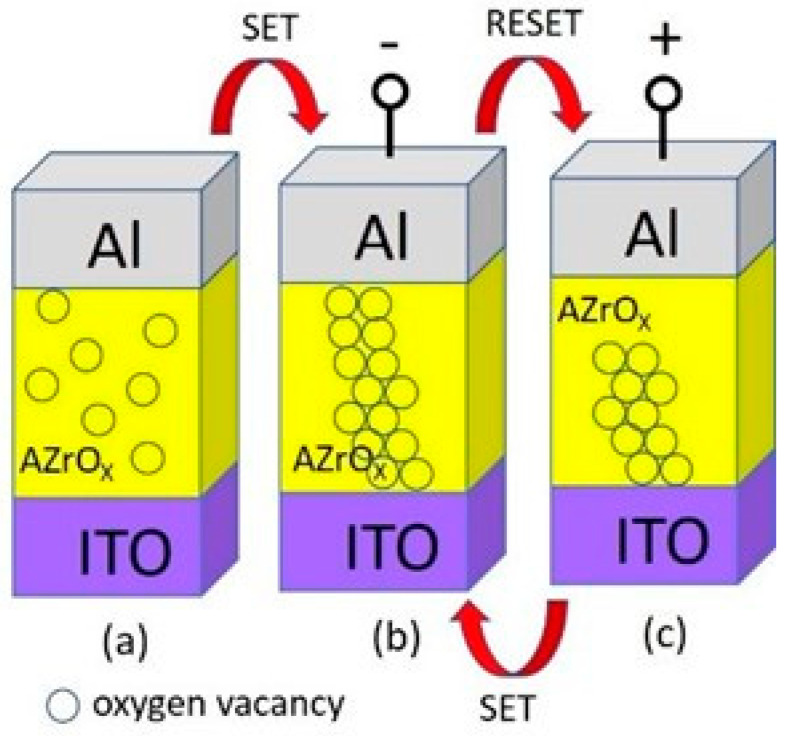
Model of the resistance switching mechanism. (**a**) pristine, (**b**) LRS, and (**c**) HRS conditions.

The physical model was proposed to describe the resistive switching mechanism of RRAM devices, as shown in Figure 8. The resistive switching mechanism of the Al/AZrO_x_/ITO is explained below. In the initial state, the Al/AZrO_x_/ITO/glass devices are not subjected to an applied bias, indicating an HRS state, as shown in Figure 8a. Figure 8b shows that negative bias is applied on the Al electrode, and the oxygen ions will move from the AZrO_x_ thin film to the ITO electrode under the action of the electric field, leaving oxygen vacancies to generate a conducting path. When the voltage reached *V_set_*, a conductive channel was formed by oxygen vacancies between the AZrO_x_ thin films and the ITO electrode. Thereafter, the device changed from the HRS to the LRS. Conversely, as shown in Figure 8c, by applying positive bias on the Al electrode, the oxygen vacancies gradually decrease, the voltage reached *V_reset_*, the conductive channel formed by oxygen vacancies between the top and bottom electrodes was broken, and the device switched from the LRS to the HRS. Basically, the conducting filament formation/rupture is caused by the oxygen ion migration.

## 3. Conclusions

The use of AZrO_x_ system (A = Mg, Sr, Ba) as the dielectric layer with MIM RRAM has been demonstrated. The HRS current values of the MZO, SZO, and BZO memory device were 7.1 × 10^−8^, 1.8 × 10^−5^, and 5.4 × 10^−7^ A, respectively. HRS current can be reduced with the less difference of atomic radius between Zr and the alkaline earth metal. The AFM image of the SZO thin film shows more particles and rougher than MZO thin film, because the atomic radius of Mg and Zr atoms differ by nearly 0%. In addition, considering that Mg has a similar electronegativity as Zr, a lower density of oxygen vacancy can be obtained from the MZO thin film. Thus, the surface roughness and the HRS current (or ON/OFF ratio) of MZO were relatively lower than those of SZO/BZO memory devices. These results can be attributed to the suitable select of element from Πa group, and the MgO_x_-stablized ZrO_x_.

## 4. Materials and Methods

**Figure 9:**
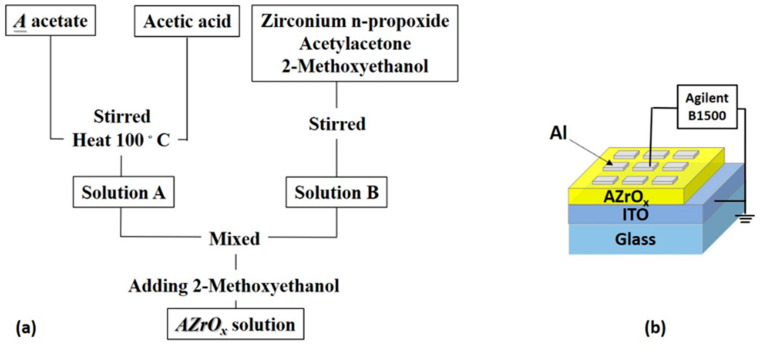
(**a**) Flowchart for the preparation of synthesized AZO_x_ solutions. (**b**) Schematic structure of the Al/AZO_x_/ITO (A = Sr, Ba, Mg) RRAM.

In the Πa (Be and Ra) group, the preparation of sol–gel precursors is not possible through the same sol–gel method, Ca (or calcium acetate) is difficult to dissolve into glacial acetic acid, and the preparation of AZrO_x_ thin film solutions involved alkaline earth metal metals, such as Mg, Sr, and Ba. First, alkaline earth metal A material (A material = 537 mg strontium acetate, 639 mg barium acetate, 356 mg magnesium acetate were added to glacial acetic acid (4 mL) and stirred at 100 ℃ to dissolve. Second, 2-methoxyethyl (4.5 mL) was mixed with zirconium (0.77 mL) and added with acetylacetone (0.47 mL) to dissolve. Finally, 0.5 M AZrO_x_ solution (A = Mg, Sr, Ba) was prepared, and solutions A and B were mixed and stirred for 24 h. The prepared AZrO_x_ solution was spin-coated on the ITO/glass substrate at 500/5000 rpm for 10/20 s, followed by Vacuum Oven at baking at 100 °C for 15 min. The synthesis of strontium zirconate (SZO), barium zirconate (BZO), and magnesium zirconate (MZO) solutions are illustrated in Figure 9a. The top electrode of the metal-insulator-metal structure was Al with an area of 3 mm^2^ with a shadow mask for electrical measurements. Figure 9b shows the schematic structure of Al/AZO_x_/ITO RRAM. We operated a semiconductor analyzer (B1500A, Agilent Technologies, Santa Clara, CA, USA) to determine the electrical properties of Al/AZO_x_/ITO storage devices. X-ray photoelectron spectroscopy (XPS) was carried out by using a PHI 5000 Versa Probe.

## Data Availability

The data that support the findings of this study are available from the corresponding author upon reasonable request.
